# Sacubitril/Valsartan Ameliorates Inflammation and Oxidative Stress in Hypertensive Heart Disease by Upregulating CAMKK2 Protein and Modulating the AMPK/AKT/GSK‐3β Axis

**DOI:** 10.1002/kjm2.70127

**Published:** 2025-10-17

**Authors:** Yan‐Jun Yang, Jiu‐Sheng Li

**Affiliations:** ^1^ Department of General Practice Tiyuzhongxin Street Community Health Service Center in Nankai District of Tianjin Tianjin China; ^2^ Department of Internal Medicine Tianjin Shuige Hospital Tianjin China

**Keywords:** CAMKK2, hypertensive heart disease, inflammation, oxidative stress, sacubitril/valsartan

## Abstract

Sacubitril/valsartan (Sac/Val) has emerged as an effective compound with myocardium‐protective functions in experimental and clinical trials for heart failure. This study investigated the function of Sac/Val in hypertensive heart disease (HHD) and explored the underlying mechanism. Spontaneous hypertensive rats (SHRs) were used as an animal model of HHD. Sac/Val administration ameliorated pathological injury, fibrosis, cell apoptosis, inflammatory cytokine production, and oxidative stress in the myocardial tissues of HHD rats. Similar findings were observed in vitro, where Sac/Val treatment reduced inflammation, oxidative stress, and fibrosis‐related markers in Ang II‐challenged H9C2 cells. Calcium/calmodulin‐dependent protein kinase kinase 2 (CAMKK2) was identified as a target of Sac/Val which was downregulated in HHD models, and CAMKK2 protein levels were restored by Sac/Val treatment. Lentiviral vector‐induced CAMKK2 knockdown reduced the phosphorylation levels of AMPKα, AKT, and GSK‐3β, thereby negating the treatment effects of Sac/Val. Further treatment with AICAR, an AMPK agonist, significantly reactivated the AMPK/AKT/GSK‐3β cascade and alleviated inflammatory injury in both models. Collectively, this study suggests that Sac/Val ameliorates inflammation and oxidative stress in HHD by restoring the CAMKK2 protein and activating the AMPK/AKT/GSK‐3β cascade.

## 1 Introduction

Persistent high blood pressure leads to changes in the structure of the left ventricle, causing hypertensive heart disease (HHD), which can eventually progress to heart failure [1]. Clinical signs of HHD include both micro‐ and macroscopic changes in the myocardium, structural adaptations, cardiac fibrosis, remodeling of the atria and ventricles of the heart, and the arterial system [2, 3]. Oxidative stress promotes aberrant redox signaling and cell injury, causing vascular damage, cardiovascular remodeling, and inflammation, all of which are important causes and consequences of hypertension [4]. The incidence of HHD and the related risk of heart failure have risen, even though there have been significant advances in the treatment and control of arterial hypertension in recent decades [5, 6]. Therefore, there is an urgent need to develop more effective treatment strategies.

A novel combination drug, sacubitril/valsartan (Sac/Val), developed by Novartis and marketed under the name Entresto, has demonstrated superior efficacy in reducing cardiovascular events and mortality as well as heart failure readmission [7]. The prodrug sacubitril is converted to sacubitrilat (LBQ657) in vivo via de‐ethylation by esterases and is known to inhibit the degradation of neprilysin, ANP, and BNP [8]. In vitro, this enzymatic conversion does not occur efficiently, leading to a lack of neprilysin inhibition, which is the intended mechanism of action [9]. The PARADIGM‐HF study (prospective comparison of ARNI with ACEI to Determine Impact on Global Mortality and morbidity in Heart Failure; NCT01035255), a randomized, double‐blind, parallel‐group trial, demonstrated that Sac/Val was more effective than enalapril in lowering the risk of death and hospitalization in patients with heart failure with reduced ejection fraction (HFrEF) [10]. This important clinical trial endorsed Sac/Val as a key angiotensin receptor–neprilysin inhibitor for HFrEF treatment [11, 12]. Additionally, Sac/Val has been beneficial in treating apparent resistant hypertension in patients with heart failure with preserved ejection fraction [13]. Despite these advantages, the effects and mechanisms of Sac/Val in HHD are not well understood.

In this study, by incorporating transcriptome sequencing analysis and bioinformatics prediction results, we identified calcium/calmodulin‐dependent protein kinase kinase kinase 2 (CAMKK2) as a promising target of Sac/Val, which is aberrantly expressed in the context of HHD. CAMKK2, a serine/threonine protein kinase, is a key component of the Ca^2+^‐calmodulin‐activated signaling pathway that plays a vital role in brain functions, such as energy metabolism [14]. When activated, CAMKK2 phosphorylates and activates several downstream kinases, including AMP‐activated protein kinase (AMPK), protein kinase B (AKT), and Ca^2+^/calmodulin‐dependent protein kinases I and IV (CaMKI and CaMKIV) [15]. Notably, CAMKK2 activation has been shown to mitigate cardiac injury in a rat model of myocardial ischemia/reperfusion injury by triggering the AMPK/AKT/glycogen synthase kinase 3β (GSK‐3β) signaling pathway [16]. It has been recently reported that the administration of Sac/Val alleviates doxorubicin‐induced cardiotoxicity by modulating the AMPKα/mTORC1 signaling pathway and suppressing oxidative stress [17]. Given these insights, this study aimed to examine whether Sac/Val can influence CAMKK2 levels and modulate the AMPK/AKT/GSK‐3β signaling pathway to reduce pathological damage in HHD.

## 2 Materials and Methods

### 2.1 Animals and Treatment

Male spontaneous hypertensive rats (SHRs), 32 weeks old, were procured from Charles River Laboratory Animal Technology Co. Ltd. (Beijing, China) and used as models for the HHD. Age‐matched male Wistar rats (Charles River) were used as controls (normal). The rats were housed under constant temperature and humidity with a 12‐h light/dark cycle and had free access to water and food.

The SHRs were orally administered Sac/Val (60 mg/kg, HY‐18204A, MedChemExpress, Monmouth Junction, NJ, USA; Sac and Val at a 1:1 M ratio) dissolved in saline containing 10% DMSO (20% SBE‐β‐CD in saline) for 10 weeks.

Before Sac/Val treatment, the rats received an intracardiac injection of lentiviral vector encapsulating short hairpin RNA (shRNA) targeting CAMKK2 or the negative control (NC) vector. Briefly, the rats were anesthetized with an intravenous injection of propofol (0.5–1.0 mg/kg/min) and secured on the operating table. An endotracheal tube was inserted and connected to a small animal ventilator set at 75 breaths/min. Upon achieving deep anesthesia, a left thoracotomy was performed between the 4th and 5th ribs to expose the heart. Using a 30‐gauge needle, sh‐CAMKK2 lentivirus (30 μL per rat, > 10^9^ TU/mL; VectorBuilder Inc., Guangzhou, Guangdong, China) was injected at two sites near the apex of the left ventricular free wall. The chest was then immediately sutured. After surgery, the rats were placed back into their cages, moved to a recovery room, and subsequently returned to the animal facility.

Additionally, two groups of SHRs were treated with the AMPK signaling activator 5‐aminoimidazole‐4‐carboxamide 1‐β‐D‐ribofuranoside (AICAR, ab146713, Abcam Inc., Cambridge, MA, USA) dissolved in phosphate‐buffered saline (PBS) or vehicle. AICAR or vehicle was administered through subcutaneous injection (0.5 g/kg/day) starting 48 h before Sac/Val treatment and continued for 10 weeks [18].

These procedures resulted in the following animal groups: Normal, HHD, SV + HHD, SV + HHD + sh‐NC, SV + HHD + sh‐CAMKK2, SV + HHD + sh‐CAMKK2 + vehicle, and SV + HHD + sh‐CAMKK2 + AICAR, each consisting of five rats. After 10 weeks, all rats were euthanized via intraperitoneal injection of 150 mg/kg pentobarbital sodium, and myocardial tissue was collected for subsequent experiments. All experimental protocols were approved by the Animal Ethics Committee of Tianjin Shuige Hospital (approval no. 20240110) and conducted following the Guide for the Care and Use of Laboratory Animals (NIH, Bethesda, MD, USA). Significant efforts have been made to reduce the number of conscious animals and their suffering.

### 2.2 Cell Culture and Treatment

Rat cardiomyocytes H9C2 (CL‐0089, Procell Life Science & Technology Co. Ltd., Wuhan, Hubei, China) were cultured in a specific medium (CM‐0089, Procell) in six‐well plates at 37°C with 5% CO_2_. H9C2 cells were infected with lentiviral vectors carrying either sh‐CAMKK2 or sh‐NC. After 72 h, stably transfected cells were identified using puromycin (2 μg/mL) post‐infection.

To mimic hypertension in vitro, H9C2 cells were exposed to Ang II (1 μM, A107852, Aladdin Biochemical Technology Co. Ltd., Shanghai, China) for 48 h. For AMPK activation in vitro, cells were treated with 10 μM AICAR for 48 h, with an equivalent volume of saline used as a control.

H9C2 cells were supplemented with 0.01 μM LBQ657 (SML2064, Sigma‐Aldrich, Merck KGaA, Darmstadt, Germany), Val (HY‐18204, MedChemExpress), or LBQ/Val in the culture medium for 48 h [19]. Saline served as the control regimen for LBQ657/Val.

### 2.3 Reverse Transcription Quantitative Polymerase Chain Reaction (RT‐qPCR)

RNA was isolated from H9C2 cells or rat myocardial tissues using the TRIzol reagent (15596026CN, Invitrogen, Thermo Fisher Scientific Inc., Waltham, MA, USA). Total RNA was then reverse‐transcribed into cDNA using the RevertAid First Strand cDNA Synthesis Kit (K16225, Thermo Fisher Scientific). The mRNA expression of CAMKK2, collagen type I alpha 1 chain (COL1A1), and collagen type III alpha 1 chain (COL3A1) was quantified using a DyNAmo HS SYBR Green qPCR Kit (F410L, Thermo Fisher Scientific). Relative expression levels normalized to glyceraldehyde‐3‐phosphate dehydrogenase (GAPDH) mRNA were determined using the 2^−ΔΔCt^ method. The primer sequences used were as follows: CAMKK2 (F): 5′‐CCTGGCCGATGAAGTTGGTA‐3′, CAMKK2 (R): 5′‐TGCGGGACATCACATTCACC‐3′; COL1A1 (F): 5′‐GGAGAGAGCATGACCGATGG‐3′, COL1A1 (R): 5′‐GGTGGGAGGGAACCAGATTG‐3′; COL3A1 (F): 5′‐TTCCTGGGAGAAATGGCGAC‐3′, COL3A1 (R): 5′‐ACCAGCTGGGCCTTTGATAC‐3′; GAPDH (F): 5′‐GCATCTTCTTGTGCAGTGCC‐3′, GAPDH (R): 5′‐GATGGTGATGGGTTTCCCGT‐3′.

### 2.4 Western Blot (WB) Analysis

H9C2 cells and rat heart tissues were lysed using radio‐immunoprecipitation assay lysis buffer (89,901; Thermo Fisher Scientific Inc.), and the total protein content was determined using a bicinchoninic acid protein assay kit (ab102536, Abcam) following the manufacturer's protocol. Protein samples were separated by 10% sodium dodecyl sulfate‐polyacrylamide gel electrophoresis and transferred onto polyvinylidene fluoride membranes (ab133411, Abcam). After blocking with 5% nonfat milk at room temperature for 1 h, the membranes were incubated overnight at 4°C with the following primary antibodies: CAMKK2 (1:500, ab135979, Abcam), AMPKα (1:1000, A0791, ABclonal Technology Co. Ltd., Wuhan, Hubei, China), p‐AMPKα (1:1000, 2535, Cell Signaling Technology, Beverly, MA, USA), AKT1 (1:1000, A17909, ABclonal), p‐AKT1 (1:1000, AP1208, ABclonal), GSK‐3β (1:1000, A6164, ABclonal), p‐GSK‐3β (1:1,000, MA5‐14873, Thermo Fisher Scientific), and GAPDH (1:5000, ab181602, Abcam). Subsequently, the membranes were probed with goat anti‐rabbit IgG H&L (1:30,000, ab205718; Abcam) at room temperature for 1 h. Immunoreactive bands were visualized using an ECL Substrate Kit (ab133406, Abcam), and band intensities normalized to GAPDH were quantified using ImageJ software.

### 2.5 Terminal Deoxynucleotidyl Transferase (TdT)‐Mediated dUTP Nick End Labeling (TUNEL)

Apoptosis was assessed according to the instructions of the TUNEL kit (C10617; Invitrogen). H9C2 cells were fixed with 4% paraformaldehyde for 15 min, permeabilized with 0.25% Triton X‐100 (P0096; Beyotime Biotechnology Co. Ltd., Shanghai, China) for 20 min, and washed twice with deionized water. Apoptosis was determined following the instructions in the TUNEL kit. Apoptotic nuclei in H9C2 cells were labeled with green fluorescein, while total nuclei were stained with 4′, 6‐diamidino‐2‐phenylindole staining solution (DAPI, ab228549, Abcam). The apoptosis rate was determined by the ratio of TUNEL‐positive to DAPI‐stained nuclei.

For rat myocardial tissues, paraffin‐embedded tissue sections were deparaffinized and rehydrated, followed by incubation with proteinase K (20 μg/mL, AM2546, Invitrogen) at room temperature for 15 min. After washing with PBS, the sections were incubated with TUNEL reaction mixture at 37°C in the dark for 30 min. Nuclei were counterstained with DAPI and observed under a fluorescence microscope. The percentage of TUNEL‐positive cells was calculated.

### 2.6 Cell Viability Detection

H9C2 cell viability was determined using Cell Counting Kit 8 (CCK‐8) (ab228554, Abcam). Briefly, cells were seeded in 96‐well plates. Each well was then added with 10 μL CCK‐8 working solution for 1 h of incubation at 37°C. Optical density was measured at 450 nm.

### 2.7 Assessment of Reactive Oxygen Species (ROS) Levels

Cellular ROS levels were measured using the fluorescent probe dihydro‐fluorescein diacetate (DCFH‐DA) (D399, Invitrogen). Briefly, H9C2 cells were incubated with 50 μM DCFH‐DA at 37°C in the dark for 30 min and washed twice with cold PBS. Fluorescence images of intracellular reactive oxygen species (ROS) were captured using a fluorescence microscope. The mean fluorescence intensity was analyzed using ImageJ software.

### 2.8 Enzyme‐Linked Immunosorbent Assay (ELISA)

Rat myocardial tissue homogenates and H9C2 cell suspensions were then prepared and collected. The concentrations of the pro‐inflammatory cytokines interleukin‐1β (IL‐1β), IL‐6, and tumor necrosis factor‐α (TNF‐α) and the anti‐inflammatory cytokine IL‐10 in the samples were determined using rat IL‐1β (ab100768, Abcam), IL‐6 (ab234570, Abcam), TNF‐α (ab46070, Abcam), and IL‐10 (ab214566, Abcam) ELISA kits. The concentrations of the oxidative stress‐related markers malondialdehyde (MDA), glutathione (GSH), and superoxide dismutase (SOD) were determined using ELISA kits for rat MDA (E‐EL‐0060, Elabscience Biotechnology Co. Ltd., Wuhan, Hubei, China), GSH (E‐EL‐0026, Elabscience), and SOD (OKEH03162, Aviva Systems Biology Corporation, San Diego, CA, USA). All procedures were performed following the manufacturer's instructions.

### 2.9 Histopathological Examination

After euthanasia, the hearts were excised and weighed, and left ventricular myocardial tissue was fixed in formaldehyde. The prepared tissue sections were deparaffinized, rehydrated, and stained with hematoxylin and eosin (HE) (C0105S, Beyotime) and Masson's trichrome staining (C0189S, Beyotime). Myocardial tissue damage observed in HE‐stained sections was quantified by examining cell spacing, cell infiltration, edema, alignment, and a histological score from 0 (none) to 4+ (100% affected), with 1+, 2+, and 3+ indicating 25%, 50%, and 75% of the affected tissue, respectively. Histological scoring was performed blinded to the treatment groups.

Collagen deposition was analyzed in four randomly selected microscopic fields per Masson‐stained section using Image‐Pro Plus 6.0. The results were expressed as collagen volume fraction (CVF, %), calculated as collagen area/total area × 100%. Stained sections were observed under a microscope.

### 2.10 Immunohistochemistry (IHC)

The prepared rat myocardial tissue sections were deparaffinized, rehydrated, soaked in heated 0.01 M citrate buffer for 20 min for antigen retrieval, and then allowed to cool to room temperature. Following this, the sections were pre‐incubated in 10% normal goat serum, washed with PBS, immersed in 3% H_2_O_2_ for 10 min, and then incubated overnight at 4°C with antibodies against p‐AMPKα (1:1000, 2535, Cell Signaling Technologies), p‐AKT1 (1:1000, AP1208, ABclonal), and p‐GSK‐3β (1:50, MA5‐14873, Thermo Fisher Scientific Inc.). Subsequently, the sections were incubated with goat anti‐rabbit IgG H&L (1:30,000, ab205718; Abcam) at 37°C for 30 min. 3,3′‐diaminobenzidine was then added at room temperature for 10 min for color development. After dehydration with ethanol, sections were cleared in xylene and sealed with neutral balsam for microscopic observation. The rate of positive staining was calculated.

### 2.11 Statistical Analysis

Data analysis was performed using Prism software (version 10.4.2; GraphPad, La Jolla, CA, USA). All values are expressed as mean ± standard error of the mean. A two‐tailed *t*‐test or one‐way analysis of variance (ANOVA) followed by Tukey's post hoc comparison was applied for qualitative analysis. For cell experiments, a minimum of three independent experiments was performed. For animal experiments, each group consisted of five rats. Statistical significance was set at *p* < 0.05.

## 3 Results

### 3.1 Sac/Val Treatment Rescues CAMKK2 Protein Levels in Animal and Cellular Models of HHD

**FIGURE 1:**
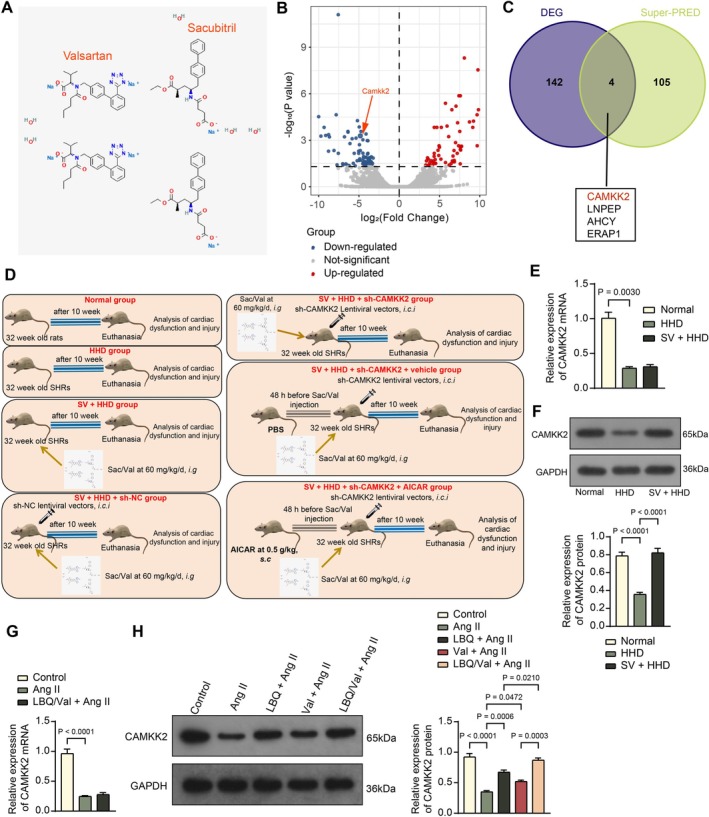
Sac/Val treatment rescues CAMKK2 protein level in animal and cellular models of HHD. (A) Chemical structure of Sac/Val. (B) DEGs (*adj. p* < 0.05) between the left ventricular tissues of SHRs (HHD models) and Wistar rats (Normal) determined using transcriptomic sequencing analysis. (C) The intersection of DEGs in the transcriptomic sequencing analysis and targets of Sac/Val in the Super‐PRED website. SHRs received daily oral gavage of Sac/Val (60 mg/kg) or an equivalent volume of normal saline for 10 weeks. (D) Flowchart for modeling and treatment of HHD in rats. (E, F) mRNA (E) and protein (F) levels of CAMKK2 in the myocardial tissue of rats in Normal, HHD, and SV + HHD groups determined by RT‐qPCR and WB analysis, respectively. (G, H) mRNA (G) and protein (H) levels of CAMKK2 in rat H9C2 cells treated with saline (Control), Ang II, or LBQ/Val + Ang II determined by RT‐qPCR and WB analysis, respectively. For animal experiments, each group consisted of five rats. For cell experiments, three independent experiments were performed. Differences were analyzed by the one‐way ANOVA (E–H).

We predicted the pharmacological targets of Sac/Val based on its chemical structure (Figure 1A) using Super‐PRED, a prediction web server for ATC codes and target prediction of compounds (https://prediction.charite.de/index.php). Additionally, transcriptomic sequencing analysis was performed to identify significantly differentially expressed genes (DEGs) (*adj. p* < 0.05) in the left ventricular tissues of SHRs (HHD models) and Wistar rats (normal) (Figure 1B). Subsequently, intersecting analysis of DEGs and the predicted pharmacological targets of Sac/Val resulted in four intersecting genes: CAMKK2, LNPEP, AHCY, and ERAP1 (Figure 1C). Among these, CAMKK2 was the most significantly differentially expressed gene (*adj. p* = 0.000600741), showing a significant reduction in expression in the left ventricle of SHRs (LogFC = −4.832423976).

HHD rats were treated with Sac/Val for 10 weeks (Figure 1D). Subsequently, RT‐qPCR and WB assays revealed decreased mRNA and protein levels of CAMKK2 in the myocardial tissue of HHD rats compared to those in normal rats. Notably, Sac/Val treatment in HHD rats significantly restored CAMKK2 protein levels without significantly influencing its mRNA expression (Figure 1E,F).

Similar trends were found in vitro, where decreased mRNA and protein levels of CAMKK2 were observed in Ang II‐treated H9C2 cells compared to saline‐treated cells. Compared to Ang II, both LBQ and Val alone increased CAMKK2 expression, and the effect of concurrent LBQ/Val treatment on the elevation of CAMKK2 protein expression was more pronounced (Figure 1G,H).

### 3.2 Sac/Val Treatment Reduces Inflammation and Oxidative Stress in HHD, Effects Negated by CAMKK2 Silencing

**FIGURE 2:**
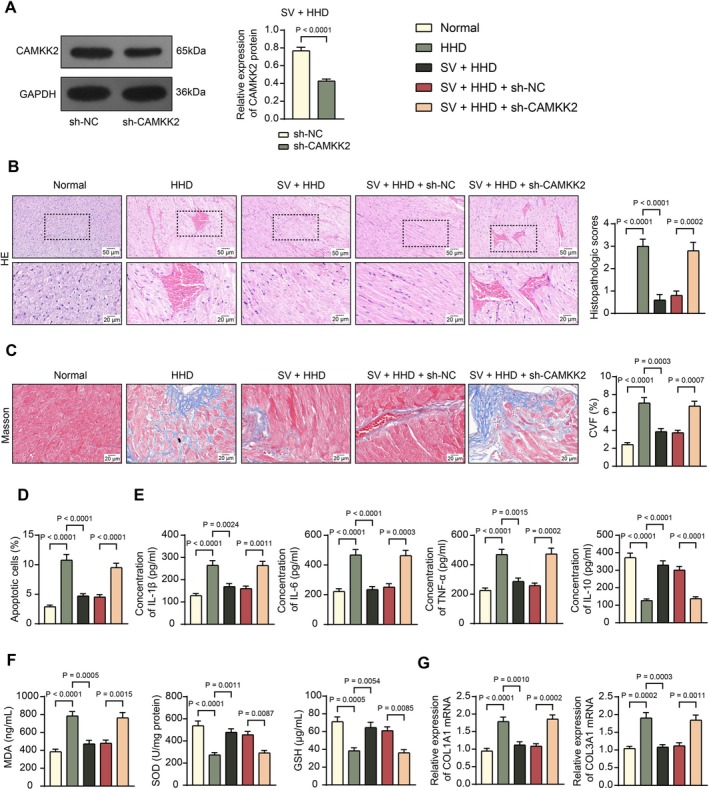
CAMKK2 silencing counteracts the anti‐inflammatory and antioxidative effects of Sac/Val in HHD. Another two groups of SHRs were pre‐administered sh‐CAMKK2 or sh‐NC before Sac/Val treatment. (A) The CAMKK2 protein level in the myocardial tissues of rats in the SV + HHD + sh‐NC and SV + HHD + sh‐CAMKK2 groups determined by WB analysis. (B) Histopathological injury scores in the myocardial tissues of rats in the Normal, HHD, SV + HHD, SV + HHD + sh‐NC, and SV + HHD + sh‐CAMKK2 groups determined by HE staining. (C) CVF in the rat myocardial tissues determined using Masson's trichrome staining. (D) Cell apoptosis in the rat myocardial tissues determined using TUNEL assay. (E) Concentrations of pro‐inflammatory cytokines IL‐1β, IL‐6, and TNF‐α as well as the anti‐inflammatory cytokine IL‐10 in the myocardial tissue homogenate determined using ELISA kits. (F) Concentrations of MDA, SOD, and GSH in the myocardial tissue homogenate determined using ELISA kits. (G) mRNA expression of COL1A1 and COL3A1 in the myocardial tissues determined using RT‐qPCR. Each group consisted of five rats. Differences were analyzed by the unpaired *t*‐test (A) or one‐way ANOVA (B–G).

Two other groups of SHRs were pre‐administered sh‐CAMKK2 or sh‐NC before Sac/Val treatment (Figure 1D). Transfection with sh‐CAMKK2 effectively reduced CAMKK2 protein levels in rat myocardial tissues (Figure 2A). HE staining revealed significant pathological injuries in the myocardial tissues of rats in the HHD group compared to those in the normal group. CVF, according to Masson's trichrome staining, was significantly increased in the HHD group. Notably, the pathological score and CVF significantly decreased following Sac/Val treatment. However, these beneficial effects of Sac/Val were negated by CAMKK2 silencing (Figure 2B,C). Moreover, the TUNEL assay revealed that cell apoptosis in rat myocardial tissues was increased in the HHD group compared to normal rats, which was inhibited by Sac/Val treatment but was restored upon CAMKK2 silencing (Figure 2D).

Similar trends were observed for inflammatory responses and oxidative stress. According to the ELISA results, compared to the normal rats, rats in the HHD group exhibited heightened concentrations of IL‐1β, IL‐6, and TNF‐α, with decreased concentrations of IL‐10 in their myocardial tissues (Figure 2E). They also exhibited increased MDA levels and decreased SOD and GSH levels (Figure 2F). However, all these alterations were reversed by Sac/Val treatment, but the effects of Sac/Val were counteracted in the presence of CAMKK2 silencing (Figure 2E,F). Additionally, RT‐qPCR revealed increased mRNA expression of COL1A1 and COL3A1 in the myocardial tissues of rats in the HHD group, which was decreased by Sac/Val treatment but restored by CAMKK2 silencing (Figure 2G). These observations revealed that CAMKK2 is involved in the anti‐inflammatory, antioxidative, and antifibrotic effects of Sac/Val in HHD.

### 3.3 CAMKK2 Silencing Counteracts the Treatment Effect of Sac/Val on Ang II‐Challenged H9C2 Cells

**FIGURE 3:**
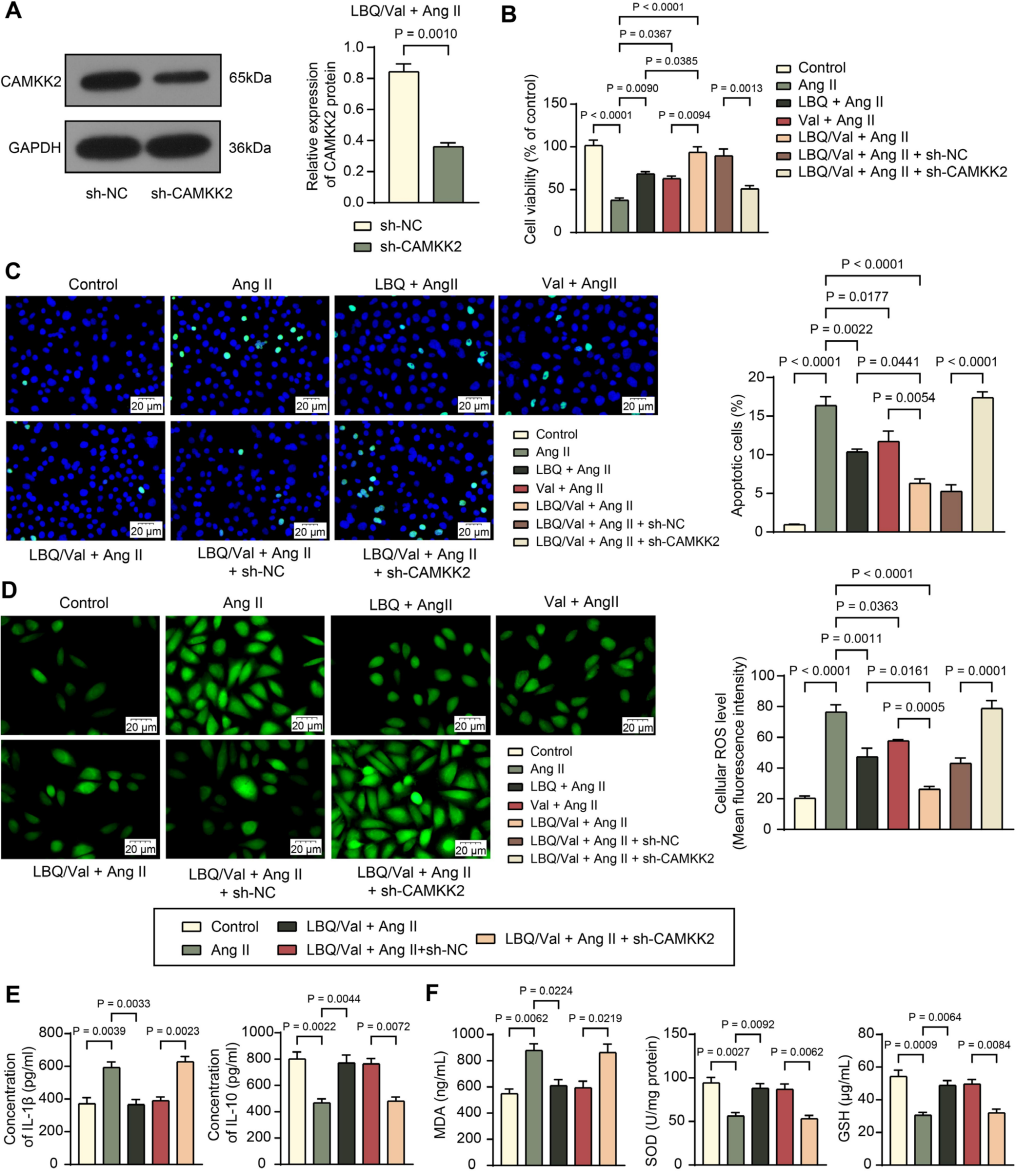
CAMKK2 silencing counteracts the treatment effect of LBQ/Val on Ang II‐challenged H9C2 cells. (A) CAMKK2 protein level in H9C2 cells in the LBQ/Val + Ang II + sh‐NC and LBQ/Val + Ang II + sh‐CAMKK2 groups determined by WB analysis. (B) Viability of H9C2 cells in the Control, Ang II, LBQ + Ang II, Val + Ang II, LBQ/Val + Ang II, LBQ/Val + Ang II + sh‐NC, and LBQ/Val + Ang II + sh‐CAMKK2 groups determined by CCK‐8 assay. (C) Apoptosis of H9C2 cells determined using TUNEL assay. (D) ROS levels in H9C2 cells determined using the DCFH‐DA probe. (E) Concentrations of IL‐1β and IL‐10 in the culture supernatant of H9C2 cells determined using ELISA kits. (F) Levels of MDA, SOD, and GSH in the culture supernatant of H9C2 cells determined using ELISA kits. Three independent experiments were performed. Differences were analyzed by the unpaired *t*‐test (A) or one‐way ANOVA (B–F).

In vitro, sh‐CAMKK2 or sh‐NC were similarly transfected into H9C2 cells before Sac/Val and Ang II treatment (Figure S1). The effective decrease in CAMKK2 protein expression was determined by WB analysis (Figure 3A). CCK‐8 assays revealed that Ang II challenge significantly decreased the viability of cells, which was restored by Sac/Val treatment but decreased again in the presence of CAMKK2 silencing (Figure 3B). According to the TUNEL assay, apoptosis of H9C2 cells was promoted by Ang II challenge, reduced by Sac/Val treatment, and increased again upon CAMKK2 silencing (Figure 3C). The DCFH‐DA probe revealed that the ROS levels in cells increased following Ang II challenge, which was decreased by Sac/Val treatment but restored by CAMKK2 knockdown (Figure 3D). It should be noted that treatment with LBQ or Val alone enhanced cell viability and repressed apoptosis and ROS levels, while their functions were not as significant as those of Sac/Val treatment.

ELISA results concerning inflammation and oxidative stress markers in the culture supernatant of H9C2 cells revealed trends consistent with those in rats. Ang II treatment upregulated the concentrations of IL‐1β and decreased the concentrations of IL‐10 (Figure 3E), accompanied by an increase in MDA levels and reduced levels of SOD and GSH (Figure 3F). These alterations were largely reversed by Sac/Val treatment. However, the anti‐inflammatory and antioxidative effects of Sac/Val on Ang II‐challenged H9C2 cells diminished following CAMKK2 knockdown.

### 3.4 Silencing of CAMKK2 Affects the AMPK/AKT/GSK‐3β Signaling Cascades

Existing evidence has demonstrated that CAMKK2 modulates the AMPK/AKT/GSK‐3β signaling pathway, thus possessing therapeutic benefits in a rat model of myocardial ischemia/reperfusion injury [16]. This prompted us to investigate whether this regulation is implicated in events modulated by CAMKK2 in the context of HHD.

**FIGURE 4:**
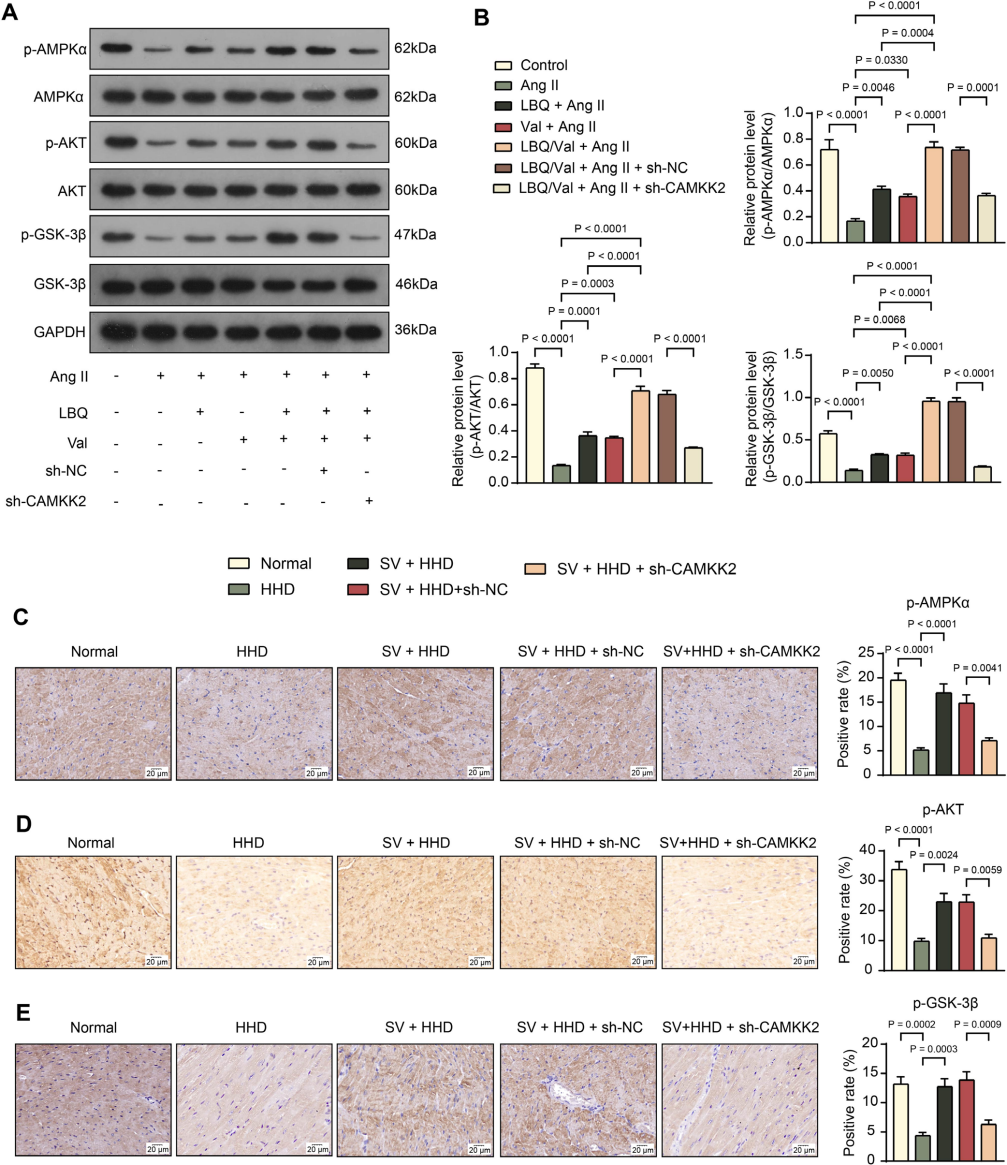
Silencing of CAMKK2 affects the AMPK/AKT/GSK‐3β signaling cascades. (A, B) The representative blots (A) and quantification (B) of phosphorylated and total protein of AMPKα, AKT, and GSK‐3β in H9C2 cells determined using WB analysis. (C–E) Phosphorylation of AMPKα (C), AKT (D), and GSK‐3β (E) in the myocardial tissue of rats determined using IHC. For cell experiments, three independent experiments were performed. For animal experiments, each group consisted of five rats. Differences were analyzed by the one‐way ANOVA (B–E).

In H9C2 cells, WB analysis revealed that the extents of AMPKα, AKT, and GSK‐3β phosphorylation were significantly decreased upon Ang II challenge. Treatment with LBQ, Val, alone or in combination, was able to promote the extents of AMPKα, AKT, and GSK‐3β phosphorylation, and the combined LBQ/Val treatment was more significant. In addition, we found that sh‐CAMKK2 decreased the extents of AMPKα, AKT, and GSK‐3β phosphorylation in the presence of LBQ/Val treatment, indicating that knockdown of CAMKK2 inhibited the activation of the AMPK signaling pathway. (Figure 4A,B).

Similarly, IHC assays revealed that positive rates of p‐AMPKα, p‐AKT, and p‐GSK‐3β were reduced, whereas Sac/Val treatment was able to promote the expression of all three in the myocardial tissues of HHD rats. Knockdown of CAMKK2 inhibited the activation of the AMPK signaling pathway, as evidenced by reduced p‐AMPKα, p‐AKT, and p‐GSK‐3β protein expression (Figure 4C–E).

### 3.5 AICAR Reactivates the AMPK Signaling Cascades and Alleviates Inflammation in HHD Rats

**FIGURE 5:**
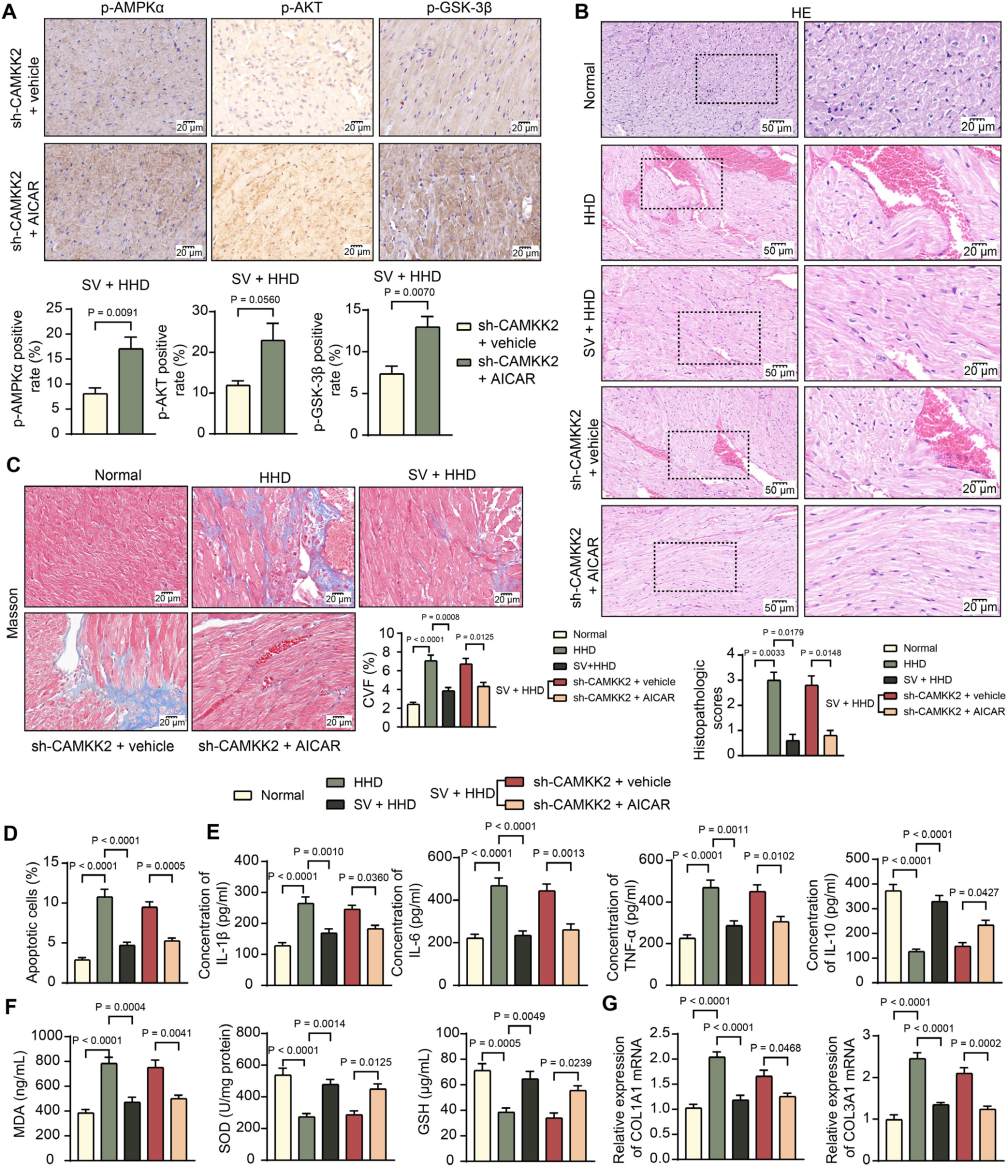
AICAR reactivates the AMPK signaling cascades and alleviates inflammation in HHD rats. SHRs stably transfected with sh‐CAMKK2 received a 10‐week treatment of AICAR (0.5 g/kg/day), an AMPK agonist, along with Sac/Val. (A) Phosphorylation of AMPKα, AKT, and GSK‐3β in the myocardial tissue of differentially treated rats determined using IHC. (B) Histopathological injury scores in the rat myocardial tissues determined by HE staining. (C) CVF in the rat myocardial tissues determined using Masson's trichrome staining. (D) Cell apoptosis in the rat myocardial tissues determined using TUNEL assay. (E) Concentrations of pro‐inflammatory cytokines IL‐1β, IL‐6, and TNF‐α as well as the anti‐inflammatory cytokine IL‐10 in the myocardial tissue homogenate determined using ELISA kits. (F) Concentrations of MDA, SOD and GSH in the myocardial tissue homogenate determined using ELISA kits. (G) mRNA expression of COL1A1 and COL3A1 in the myocardial tissues determined using RT‐qPCR. Each group consisted of five rats. Differences were analyzed by the unpaired *t*‐test (A) or one‐way ANOVA (B–G).

Two other groups of SHRs stably transfected with sh‐CAMKK2 received a 10‐week treatment with AICAR, an AMPK agonist, along with Sac/Val (Figure 1D). AICAR treatment effectively increased AMPKα, AKT, and GSK‐3β phosphorylation in rat myocardial tissues (Figure 5A). This led to reduced pathological scores and CVF according to histopathological staining (Figure 5B,C). Moreover, apoptosis within the myocardial tissues was alleviated by AICAR treatment (Figure 5D). Regarding the inflammatory response and oxidative stress, AICAR treatment decreased the concentrations of IL‐1β, IL‐6, and TNF‐α, while enhancing the concentrations of IL‐10 in the myocardial tissues (Figure 5E), accompanied by decreased MDA levels and increased levels of SOD and GSH (Figure 5F). Moreover, the mRNA expression levels of COL1A1 and COL3A1 in the myocardial tissues of rats were decreased by AICAR treatment (Figure 5G). This evidence suggests that reactivation of the AMPK/AKT/GSK‐3β signaling cascade alleviates inflammation, oxidative stress, and fibrosis in HHD rats, but does not completely return to the normal level.

### 3.6 AICAR Alleviates H9C2 Cell Injury in the Presence of CAMKK2 Silencing

**FIGURE 6:**
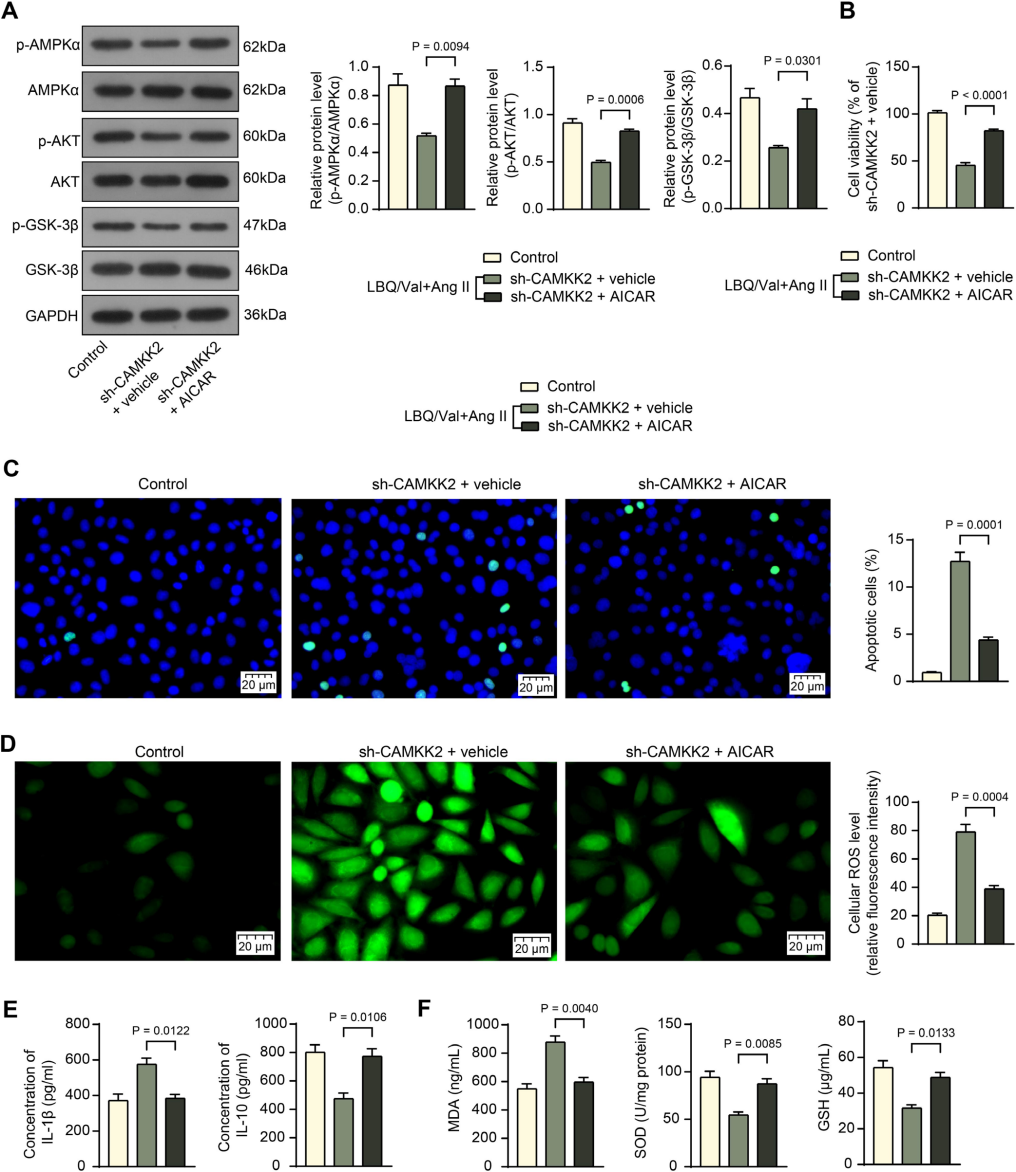
AICAR alleviates H9C2 cell injury in the presence of CAMKK2 silencing. H9C2 cells stably transfected with sh‐CAMKK2 were administered AICAR (10 μM) along with Ang II (1 μM) and LBQ/Val (0.01 μM). (A) Levels of phosphorylated and total protein of AMPKα, AKT, and GSK‐3β in H9C2 cells determined using WB analysis. (B) Viability of H9C2 cells determined by CCK‐8 assay. (C) Apoptosis of H9C2 cells determined using TUNEL assay. (D) ROS levels in H9C2 cells determined using the DCFH‐DA probe. (E) concentrations of IL‐1β and IL‐10 in the culture supernatant of H9C2 cells determined using ELISA kits. (F) Levels of MDA, SOD, and GSH in the culture supernatant of H9C2 cells determined using ELISA kits. Three independent experiments were performed. Differences were analyzed by the one‐way ANOVA (A–F).

Analogously, H9C2 cells stably transfected with sh‐CAMKK2 were administered AICAR, Ang II, and Sac/Val. AICAR treatment also increased the phosphorylation of AMPKα, AKT, and GSK‐3β in H9C2 cells (Figure 6A). This led to increased cell viability (Figure 6B), whereas it reduced cell apoptosis (Figure 6C), accompanied by a decrease in the cellular ROS levels (Figure 6D). Mirroring the findings in vivo, the AICAR treatment also decreased the levels of IL‐1β while enhancing the IL‐10 levels in the culture supernatant of H9C2 cells (Figure 6E), as well as it reduced the levels of MDA while restoring the levels of SOD and GSH (Figure 6F). When compared to the Control group without Ang II treatment, the treatment of AICAR greatly alleviated the cellular damage, but did not completely recover.

## 4 Discussion

With life expectancy and the prevalence of hypertension predicted to rise in the coming decade, HHD is likely to become increasingly significant in the pathophysiology of cardiovascular disease [3]. While Sac/Val has been extensively studied for its therapeutic efficacy in the management of heart failure, its biological function in HHD, particularly the functional mechanisms, remains largely undefined. In this study, the authors validate that Sac/Val possesses anti‐inflammatory and antioxidative effects in the context of HHD and report the involvement of CAMKK2‐mediated AMPK/AKT/GSK‐3β signaling cascades in these events.

As introduced above, administration of Sac/Val exhibited a better therapeutic effect than the conventional angiotensin‐converting–enzyme inhibitor enalapril in alleviating heart failure‐associated symptoms, hospitalization rates, and mortality in patients with HFrEF [10]. The outstanding myocardium‐protective function of Sac/Val has also been highlighted in clinical trials of acute myocardial infarction [20, 21]. Concerning the role of Sac/Val in the context of HHD, several animal experiments have been conducted with encouraging conclusions. For instance, a 3‐month treatment of Sac/Val has been found to significantly improve endothelium‐dependent hyperpolarization‐mediated response in SHRs [22]. In high‐salt‐loaded SHRs, the Sac/Val treatment significantly decreased the blood pressure of animals, mitigating cardiac hypertrophy and inflammation, coronary arterial remodeling, and vascular endothelial dysfunction [23]. Moreover, Val + Sacubitrilat (LBQ657) was also superior to LBQ657 or Val in improving the electrical and structural remodeling of HL‐1 cells [24]. The anti‐hypertensive effect of Sac/Val has also been demonstrated in randomized clinical trials [25]. Consistent with these publications, we verified that Sac/Val administration ameliorated pathological injuries, fibrosis, cell apoptosis, inflammatory cytokine production, and oxidative stress in the myocardial tissues of SHRs, with parallel anti‐inflammatory, antioxidative, anti‐fibrotic, and anti‐apoptotic effects of Sac/Val found in Ang II‐exposed H9C2 cells. These observations provide novel evidence supporting Sac/Val as a promising and effective regimen for HHD management.

In a rodent model with myocardial infarction, Sac/Val treatment increased cardiac function and decreased myocardial fibrosis through the downregulation of exosomal microRNA‐181a [26]. Moreover, Sac/Val mitigated myocardial injury and inflammation by suppressing the TAK1/JNK signaling pathway and reducing the NLR pyrin family domain‐containing 3‐induced pyroptosis [27]. In this study, the intersection analysis of pharmacological targets of Sac/Val and aberrantly expressed genes in the left ventricular tissues of HHD rats revealed CAMKK2 as a promising target. CAMKK2 is crucial for maintaining whole‐body energy homeostasis [28]. Its expression has been found to decrease in the left ventricle of aged rats, along with a deactivation of the AMPK signaling pathway [29]. Kim et al. identified tetrahydrobiopterin as an endogenous activator of CAMKK2, which rescued mitochondrial and cardiac dysfunction in rats with diabetic cardiomyopathy [30]. AMPK is an energy sensor that fulfills pleiotropic cardioprotective effects, with its dysregulation implicated in the progression of HHD or heart failure [31, 32]. Activation of the CAMKK2‐AMPK axis has also been associated with lipid metabolism and ATP synthesis, which restricts cardiac hypertrophy and improves heart function [33]. Notably, CAMKK2 phosphorylates the catalytic subunit of AMPK at Thr172, resulting in large enzymatic activation in cultured cells [34, 35]. This AMPK/PI3K/GSK‐3β cascade has been demonstrated to activate the expression of nuclear factor erythroid 2‐related factor 2, leading to reduced inflammation, oxidative stress, and cell apoptosis in ischemia/reperfusion injury in the myocardium [16] as well as in the brain [36, 37]. In this study, we validated that the Sac/Val treatment increased CAMKK2 protein level without altering its mRNA expression, leading to increased phosphorylation levels of AMPKα, AKT, and GSK‐3β. Furthermore, the CAMKK2 knockdown reduced AMPKα/AKT/GSK‐3β phosphorylation, negating the treatment effects of Sac/Val. Further treatment of AICAR, an AMPK agonist, significantly reactivated the AMPK/AKT/GSK‐3β cascade and alleviated inflammatory injuries in both animal and cellular models. These observations indicate that the CAMKK2/AMPKα/AKT/GSK‐3β axis is implicated in the protective effects of Sac/Val against HHD.

Our study has several limitations that should be noted. Firstly, sac inhibited neprilysin, leading to increased levels of natriuretic peptides [38]. We proposed that these peptides might influence intracellular calcium dynamics, which may activate CAMKK2. Verifying the proposed mechanism might be the next direction. In addition, future studies (e.g., cellular uptake assays, target engagement studies) are needed to validate the hypothesis.

## 5 Conclusion

**FIGURE 7:**
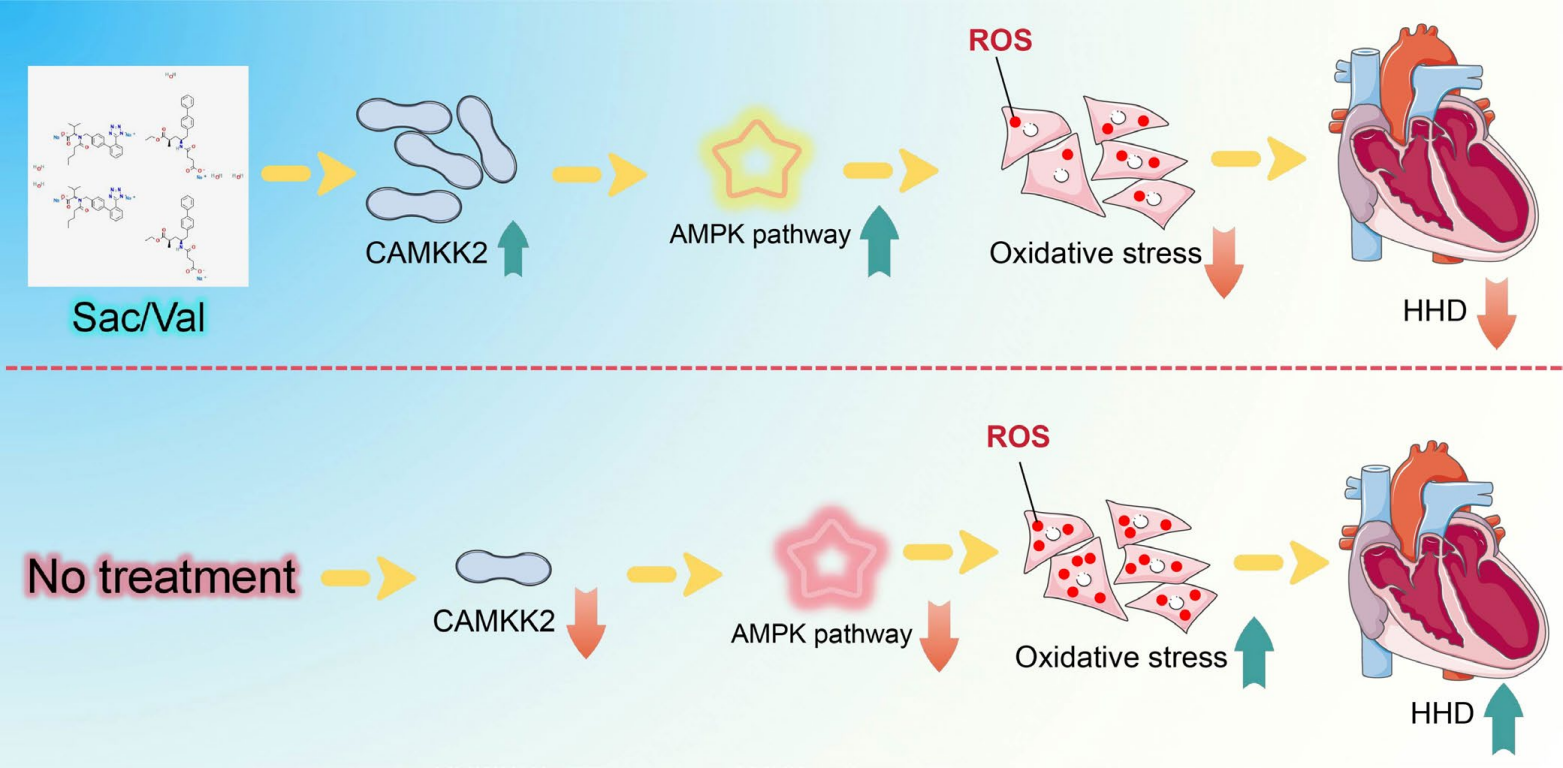
Schematic model of this study. Sac/Val ameliorates HHD by inhibiting oxidative stress through activation of CAMKK2‐mediated AMPK signaling.

In conclusion, while the myocardium‐protective role of Sac/Val is not that new, this study provides a novel understanding that Sac/Val alleviates inflammation, oxidative stress, cell apoptosis, and pathological injury in HHD by upregulating CAMKK2 and mediating AMPK/AKT‐dependent GSK‐3β deactivation (Figure 7). These observations may provide new insights into the application of Sac/Val as an effective regimen for the management of HHD.

## Conflicts of Interest

The authors declare no conflicts of interest.

## Supporting information


**Figure S1:** Flowchart of in vitro H9C2 cell treatment.

## Data Availability

The data that support the findings of this study are available on request from the corresponding author. The data are not publicly available due to privacy or ethical restrictions.
