# Supplementary figures and images for: Sacubitril/Valsartan Ameliorates Inflammation and Oxidative Stress in Hypertensive Heart Disease by Upregulating CAMKK2 Protein and Modulating the AMPK/AKT/GSK‐3β Axis
Source: Kaohsiung J Med Sci. 2025 Oct 17;42(4):e70127. doi: 10.1002/kjm2.70127 (PMC13147946; doi:10.1002/kjm2.70127)

**Supplementary Fig 1**

**
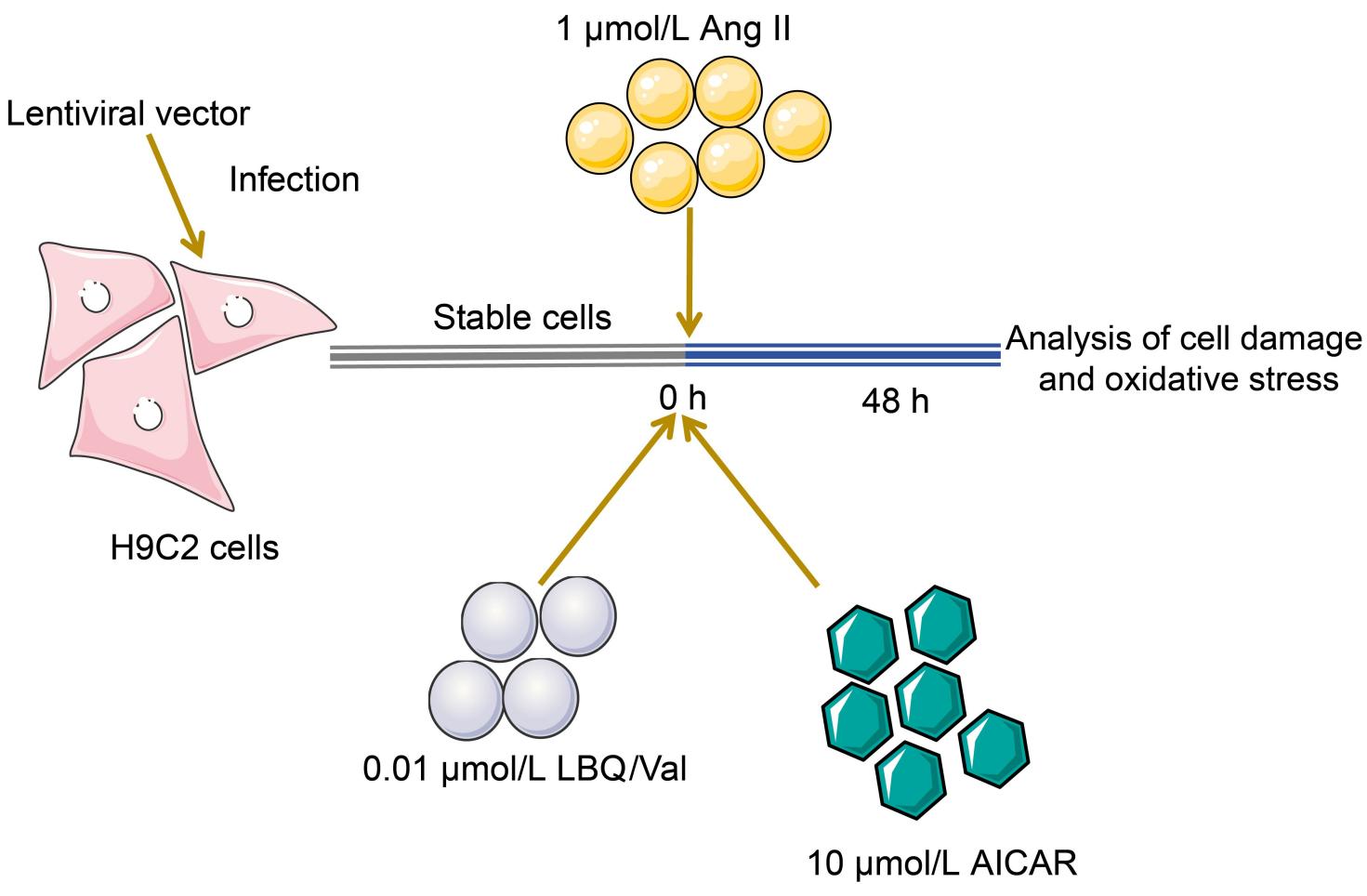
**

**Supplementary Fig 1** Flowchart of *in vitro* H9C2 cell treatment.

Supplement: Supplementary file 1 — Figure S1: Flowchart of in vitro H9C2 cell treatment. [file KJM2-42-e70127-s001.docx]
